# Endothelial-specific insulin receptor substrate-1 overexpression worsens neonatal hypoxic-ischemic brain injury via mTOR-mediated tight junction disassembly

**DOI:** 10.1038/s41420-021-00548-3

**Published:** 2021-06-29

**Authors:** Yi-Fang Tu, Si-Tse Jiang, Chi-Wu Chiang, Li-Ching Chen, Chao-Ching Huang

**Affiliations:** 1 Department of Pediatrics, National Cheng Kung University Hospital, College of Medicine, National Cheng Kung University, Tainan, Taiwan; 2 Institute of Clinical Medicine, College of Medicine, National Cheng Kung University, Tainan, Taiwan; 3 National Laboratory Animal Center, National Applied Research Laboratories, Taipei, Taiwan; 4 Institute of Molecular Medicine, College of Medicine, National Cheng Kung University, Tainan, Taiwan; 5 TMU Research Center of Cancer Translational Medicine, Taipei Medical University, Taipei, Taiwan

**Keywords:** Neuro-vascular interactions, Hypoxic-ischaemic encephalopathy, Paediatric research

## Abstract

Hypoxic-ischemic (HI) encephalopathy is the major cause of mortality and disability in newborns. The neurovascular unit is a major target of acute and chronic brain injury, and therapies that protect simultaneously both neurons and vascular endothelial cells from neonatal HI injury are in demand. Insulin receptors and its key downstream molecule-insulin receptor substrate −1 (IRS-1) are potential neuroprotective targets and expressed both in neuron and endothelial cells. To investigate whether IRS-1 can act similarly in neurons and vascular endothelial cells in protecting neurovascular units and brain form HI injury, we found that neuron-specific IRS-1 transgenic rats showed reduced neurovascular injury and infarct volumes, whereas endothelial-specific IRS-1 transgenic rats showed increased blood-brain barrier (BBB) disruption and exaggerated neurovascular injury after neonatal HI brain injury. Endothelial-specific IRS-1 overexpression increased vascular permeability and disassembled the tight junction protein (zonula occludens-1) complex. Inhibition of mammalian target of rapamycin (mTOR) by rapamycin preserved tight junction proteins and attenuated BBB leakage and neuronal apoptosis after HI in the endothelial-specific IRS-1 transgenic pups. Together, our findings suggested that neuronal and endothelial IRS-1 had opposite effects on the neurovascular integrity and damage after neonatal HI brain injury and that endothelial IRS-1 worsens neurovascular integrity after HI via mTOR-mediated tight junction protein disassembly.

## Introduction

Perinatal asphyxia occurs in 1–1.5% of live births in developed countries, and higher in developing countries [1, 2]. Nearly 50% of infants who have severe perinatal asphyxia develop hypoxic-ischemia (HI) encephalopathy, which is the major cause of mortality and of disability in newborns worldwide [2]. In recent two decades, early therapeutic hypothermia is well established as a standard treatment for infants with HI encephalopathy but is only partially effective with an absolute reduction from 61% to 46% of mortality and of disability [3]. However, nearly half of infants die or survive with significant neurological disabilities at 18–22 months of age despite receiving therapeutic hypothermia treatment [3, 4]. Therefore, development of other therapeutic interventions for newborns who suffer from HI encephalopathy is needed.

The neurovascular unit, consisting of neurons, microvessels, and other surrounding cells, is a major target of acute and chronic brain injury [5–8]. Dysfunction of the neurovascular unit can disrupt microcirculation and promote the progression of acute and chronic neurological diseases [9–11]. Neurovascular damage may occur in the early stage of neurological diseases, even before the onset of neuronal death [12]. Communication between the nervous and vascular systems in the neurovascular unit is required for maintaining the blood-brain barrier (BBB) integrity and for promoting neural function and regeneration [5, 8]. Therefore, it is better to develop brain protective therapies to target molecules or pathways that can act positively both on neurons and vessels simultaneously in the neurovascular unit.

The brain is now regarded as an insulin-sensitive organ with a widespread expression of the insulin receptor in the cerebral cortex, hippocampus, amygdala, hypothalamus, and cerebellum [13]. Insulin receptor signaling in the brain is important for neuronal development, dendritic outgrowth, synaptic plasticity, neuronal survival, circuit development, and cognitive function [13, 14]. Evidence has demonstrated impaired insulin receptor signaling or brain insulin resistance occurs in several neurological disorders. Insulin receptor substrate −1 (IRS-1) is a key protein in transducing signaling from cell surface insulin receptors to downstream signaling pathways. Both insulin receptors and IRS-1 are ubiquitously expressed in different cell types, including neurons and endothelial cells [15, 16]. Our previous study revealed that increased IRS-1 expression levels in the neurovascular unit by a moderate dietary restriction provided neuroprotection against brain damage after hypoxic-ischemia (HI) in rat pups [17]. Dietary restriction-mediated neurovascular protection after HI was mediated through an IRS-1/Akt-mediated p53 downregulation [17, 18]. These findings suggest that upregulation of IRS-1 in the neurovascular unit may be an important therapeutic strategy against HI brain injury.

However, little is known on whether IRS-1 plays a similar role in neuronal cells and vascular endothelial cells of the neurovascular unit in neuroprotection against neonatal HI brain injury. In this study, we used transgenic rat pups in which IRS-1 was respectively overexpressed in the neuronal or the endothelial cells to investigate the respective effect and the underlying mechanism of IRS-1-regulated neurovascular integrity after neonatal HI brain injury.

## Results

### IRS-1 overexpression reduced BBB disruption and neuronal apoptosis after neonatal HI injury

**Fig. 1:**
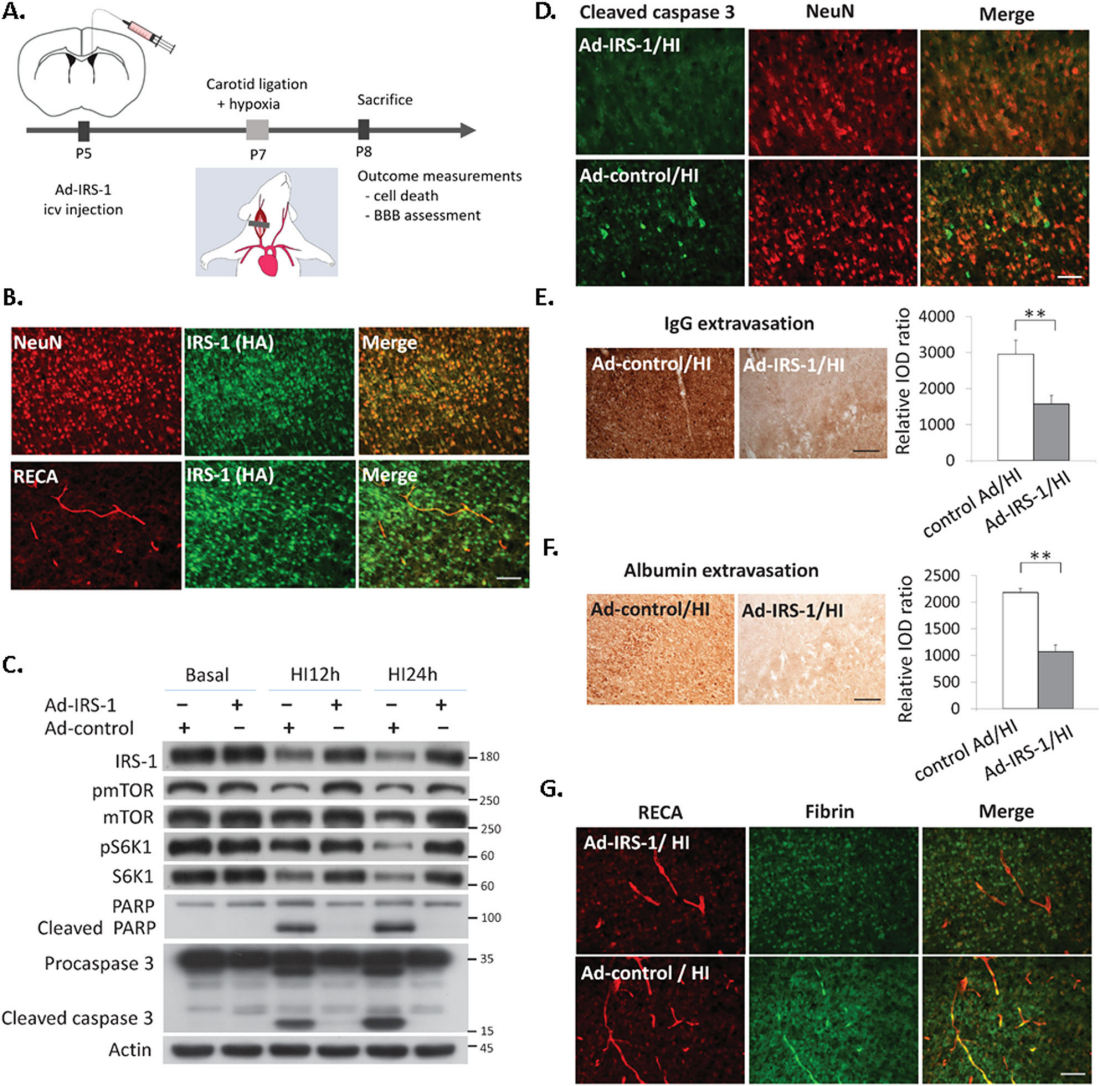
Ectopic expression of IRS-1 by recombinant adenovirus-IRS-1 protected against neurovascular injury after hypoxic-ischemia. **A** Diagram of the experimental procedure: rat pups received intracerebroventricular (icv) injection of recombinant adenovirus IRS-1 (Ad-IRS-1) or control adenovirus (Ad-control) on postnatal (P) day 5, had hypoxic-ischemic (HI) brain injury on P7, and scarified at 24 h after HI for outcome measurements. **B** Expression of HA-tagged IRS-1 mediated by Ad-IRS-1 on NeuN (+) neurons and RECA(+) endothelium cells in the cerebral cortex of P7 rat pups. *N* = 4, Scale bar: 50 μm. **C** The Ad-IRS-1-treated rats showed upregulate the expression of IRS-1 in the brain, and reduced cleaved-PARP, and –capsease 3 after HI compared to the Ad-control treated rats. *N* = 5. **D** The expression of cleaved caspases-3 in NeuN (+) neurons was obvious in the Ad-control-infected rats at 24 h after HI compared to the Ad-IRS-1-infectedrats. *N* = 4, Scale bar: 50 μm. **E**, **F** Representative images illustrate immunoglobulin (IgG) and albumin extravasation in the cerebral cortex at 24 h after HI between the Ad-IRS-1-infected and Ad-control-infected rats, and the relative integrated optic density (IOD) of IgG (*N* = 4, *P* = 0.001, *t* = 6.305) and albumin (*N* = 4, *P* < 0.001, *t* = 14.644) signals was compared. Scale bar: 125 um. Independent *t*-test was used for statistical analysis. All data are presented as mean ± SD. ** *P* < 0.01. **G** The Ad-IRS-1 rats had more perivascular fibrin deposits than the Ad-control rats at 24 h after HI. *N* = 4, Scale bar: 50 μm.

Overexpression of IRS-1 was induced by intracerebroventricular injection of recombinant adenovirus harboring HA-tagged IRS-1 (Ad-IRS-1) (Fig. 1A). The rat pups infected with Ad-IRS-1 showed the HA-tagged IRS-1protein was overexpressed in the NeuN (+) neurons and RECA (+) endothelium cells (Fig. 1B). At 24 h after HI, the Ad-IRS-1-infected pups showed decreased levels of the cleaved Poly(ADP-ribose) polymerase (PARP) and cleaved caspase 3 protein (Fig. 1C), and that the level of cleaved caspase 3 was reduced in Ad-IRS-1-infected NeuN (+) neurons compared to that of the control adenovirus (Ad-control) infected pups (Fig. 1D). The Ad-IRS-1-infected pups also showed significant decreases in levels of extravasation of immunoglobulin G (IgG), albumin, and fibrin, which are hallmarks of BBB disruption (Fig. 1E–G). These findings suggested that IRS-1 overexpression by Ad-IRS-1 infection reduced neuronal and vascular injury after HI.

### Transgenic rats with neuronal IRS-1 overexpression had reduced neurovascular injury and decreased brain damage after HI

**Fig. 2:**
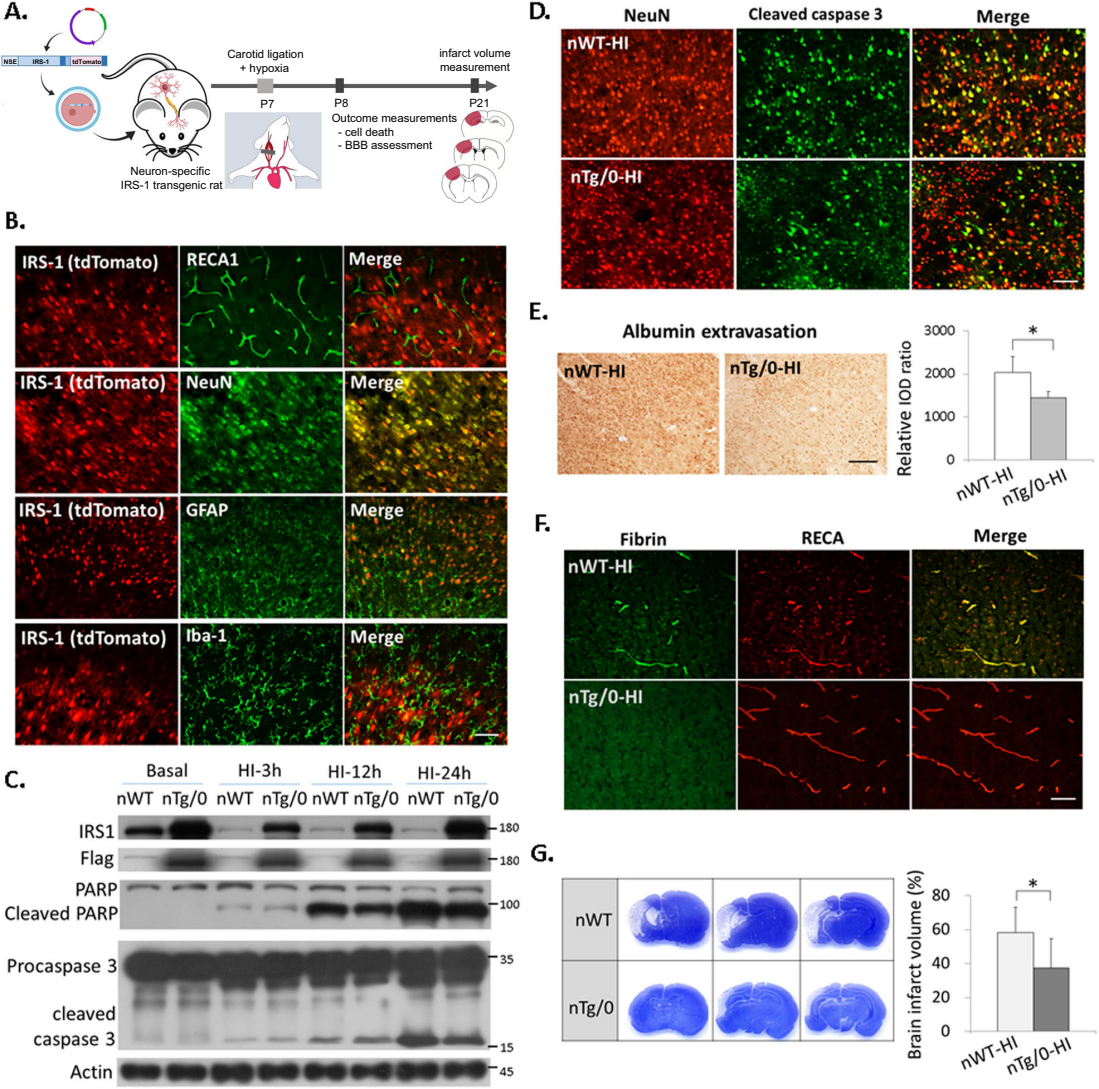
Neuron-specific IRS-1 transgenic rats had reduced neurovascular injury and brain damage after HI. **A** Diagram of the experimental procedure in the neuron-specific IRS-1 transgenic rats (nTg/0) and wild-type littermates (nWT): HI on P7, and outcome assessments on P8 and P21. The transgene contains neuron-specific promoter (NSE), full-length IRS-1 tagged with 3x Flag and IRES-directed tdTomato. **B** The IRS-1/tdTomato were expressed specifically on NeuN (+) neurons, and not on RECA(+) endothelium cells, GFAP(+) astrocytes or Iba-1(+) microglia in the cerebral cortex of P7 rat pups. Scale bar: 50 μm. **C** The nTg/0 rats showed upregulated IRS-1 in the brain, and reduced cleaved-PARP, and –capsease 3 after HI compared to the nWT rats. *N* = 4 **D** The cleaved caspase 3 (+) and NeuN (+) apoptotic neurons were reduced in the nTg/0 rats than that in the nWT rats at 24 h after HI. *N* = 4, Scale bar: 50 μm. **E** Representative images illustrate albumin extravasation in the cerebral cortex at 24 h after HI between the nTg/0 than nWT rats, and the relative integrated optic density (IOD) of albumin signal was quantified and compared between groups. Scale bar: 125um. Independent *t*-test was used for statistical analysis (*N* = 5, *P* = 0.017, *t* = 3.402). The data are presented as mean ± SD. * *P* < 0.05. **F** The nTg/0 rats had less perivascular fibrin deposits than that of the nWT rats at 24 h after HI. *N* = 4, Scale bar: 50 μm. **G** The brain infarct volume on P21 (14 days after HI) was quantified and compared between the nTg/0 and nWT rats. Independent *t*-test was used for statistical analysis (*N* = 10, *P* = 0.01, *t* = 2.888). The data are presented as mean ± SD. * *P* < 0.05.

**Fig. 3:**
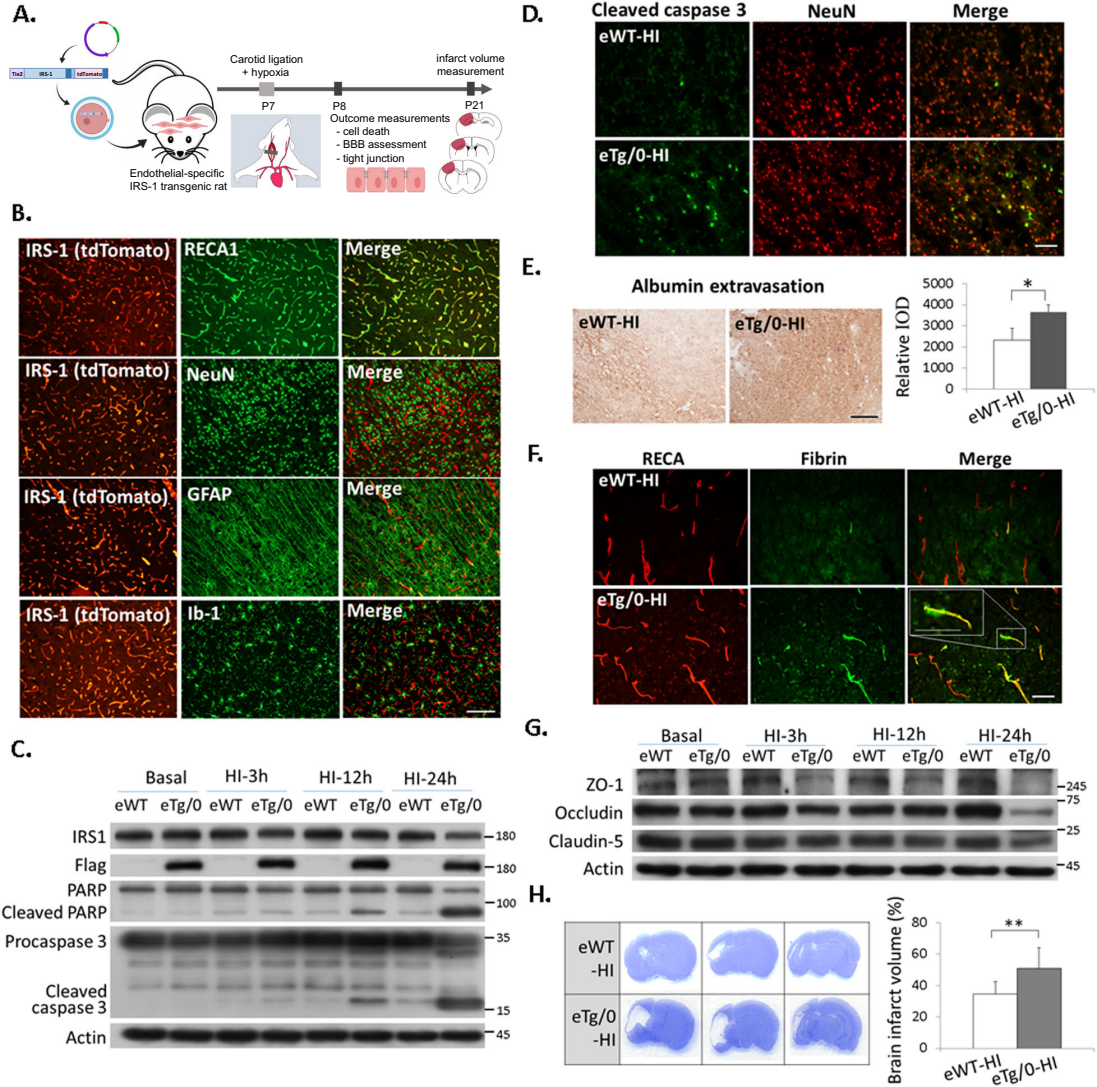
Endothelial-specific IRS-1 transgenic rats showed augmented neurovascular injury and brain damage after HI. **A** Diagram of the experimental procedure in the endothelial-specific IRS-1 transgenic rats (eTg/0) and wild-type littermates (eWT) rats: HI on P7, and outcome assessments on P8 and P21. The transgene contains endothelial-specific promoter (Tie2), full-length IRS-1 tagged with 3x Flag and IRES-directed tdTomato. **B** IRS-1/tdTomato was expressed specifically on RECA (+) endothelium cells, and not on NeuN(+) neurons, GFAP(+) astrocytes, or Iba-1(+) microglia in the cerebral cortex of P7 rat pups. Scale bar: 50 μm. **C** The eTg/0 rats had increased levels of cleaved-PARP, and –capsease 3 after HI compared to the eWT rats. *N* = 4. **D** Increased cleaved caspase 3 (+) and NeuN (+) apoptotic neurons were noted in the eTg/0 rats compared to that in the eWT rats at 24 h after HI. Scale bar: 50 μm. *N* = 4. **E** Representative images illustrate albumin extravasation in the cerebral cortex at 24 h after HI between eTg/0 and eWT rats, and the relative integrated optic density (IOD) of albumin signal was quantified. Scale bar: 125 μm. Independent *t*-test was used for statistical analysis (*N* = 6, *P* = 0.001, *t* = −5.040). The data are presented as mean ± SD. * *P* < 0.05. **F** The extravascular fibrin is more in the eTg/0 rats compared to that in the eWT rats at 24 h after HI. Scale bar: 50 μm. Inset scale bar: 50um. *N* = 4. **G** Immunoblotting analysis of tight junction proteins in the brain of eTg/0 rats. *N* = 4. **H** The brain infarct volume was compared between eTg/0 and eWT rats. Independent *t*-test was used for statistical analysis (*N* = 12, *P* = 0.002, *t* = −3.638). The data are presented as mean ± SD. ** *P* < 0.01.

To further elucidate whether there are differential effects on neuroprotection against HI by overexpression of IRS-1 in the neuron compared to that in the vascular endothelial cells, the transgenic rats with neuronal-specific overexpression of IRS-1 (nTg/0) and transgenic rats with vascular endothelium specific overexpression of IRS-1 (eTg/0) were generated, respectively (Figs. 2A, 3A). In the nTg/0 rat pups, the overexpressed Flag-tagged IRS-1(IRS-1-Flag) co-localized only with NeuN (+) neurons, but not with RECA (+) endothelial cells, GFAP (+) astrocytes, or Iba-1(+) microglia (Fig. 2B). At 24 h after HI, the nTg/0 pups had decreased levels of both cleaved-PARP and -caspase 3 in the cortex (Fig. 2C) and reduced levels of cleaved caspase 3 in neurons compared to that of the wild-type littermates (nWT) (Fig. 2D). The nTg/0 pups also showed significantly attenuated extravasation of albumin and fibrin after HI (Fig. 2E, F). Furthermore, the nTg/0 pups had significantly reduced brain infarct volume than that of the nWT pups at 14 days after HI (Fig. 2G). This neuroprotective effect was similar in another linage of neuron-specific IRS-1 transgenic rat pups (Supplementary Fig 1).

### Transgenic rat pups with endothelial IRS-1 overexpression had reduced tight junction proteins, increased neurovascular injury and increased brain damage after HI

In the eTg/0 rat pups, the overexpressed IRS-1-Flag co-localized only with RECA (+) endothelial cells, but not with NeuN (+) neurons, GFAP (+) astrocytes, or Iba-1(+) microglia (Fig. 3B, Supplementary Fig 2). At 24 h after HI, the eTg/0 pups had augmented cleaved-PARP and -caspase 3 protein expression in the cortex (Fig. 3C), and showed increased number of cleaved-caspase 3 (+) neurons compared to that of wild-type littermates (eWT) (Fig. 3D). The eTg/0 pups also had augmented BBB disruption as evidenced by more extravasation of albumin and fibrin than that of the eWT pups (Fig. 3E, F). We examined the tight junction proteins, including zonula occludens-1 (ZO-1), claudin-5 and occluding, which contribute to the BBB integrity, and found that all of these tight junction proteins were reduced in eTg/0 pups at 24 h after HI. In particular, ZO-1 levels were markedly reduced in the eTg/0 pups at 3 h, 12 h, and especially 24 h after HI (Fig. 3G). In addition, the eTg/0 pups showed significantly increased brain infarct volume after HI than the eWT pups (Fig. 3H).

### Endothelial IRS-1 overexpression increased vascular permeability and disassembled ZO-1 expression in the tight junction

**Fig. 4:**
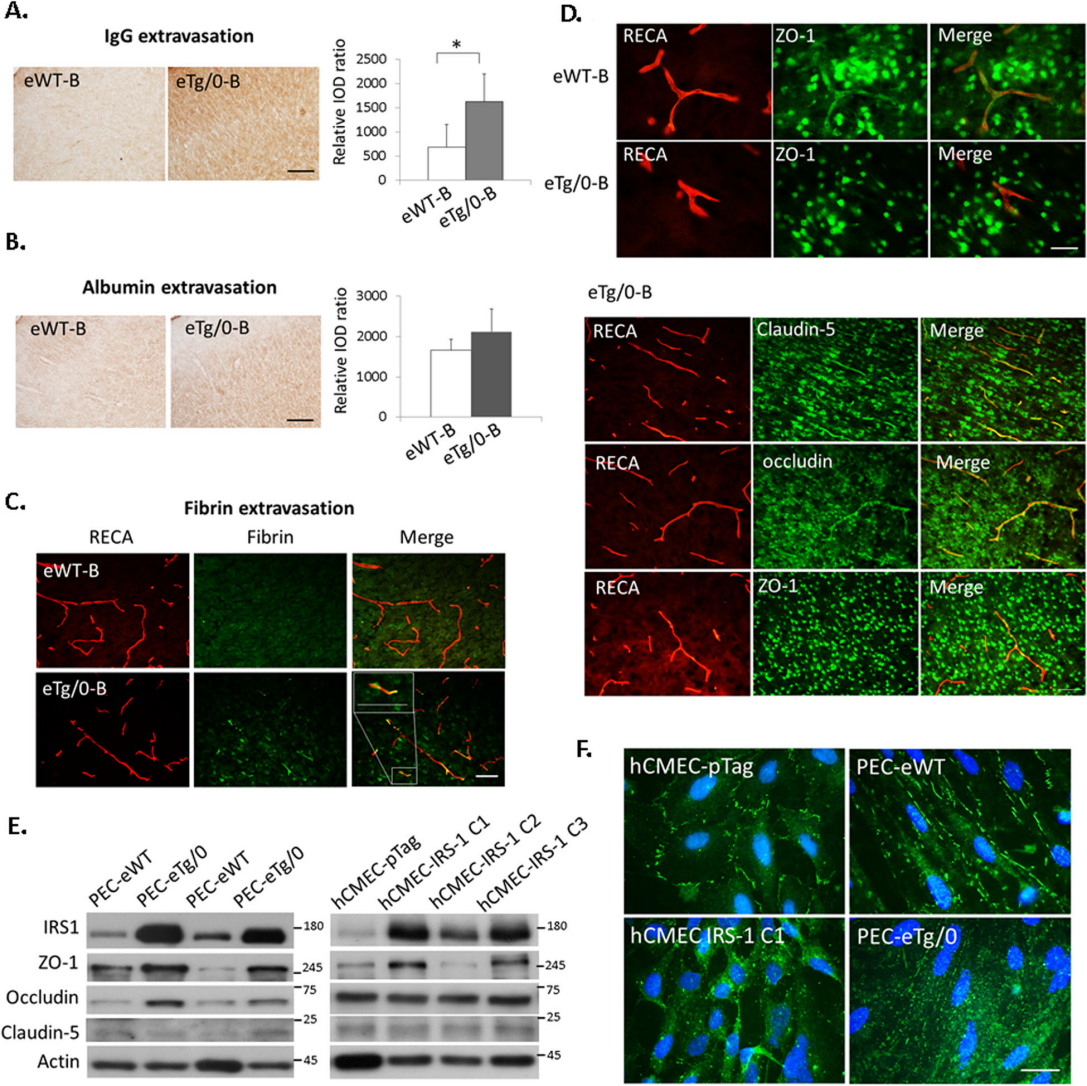
Disturbed BBB functions and aberrant distribution of tight junction protein ZO-1 by IRS-1 overexpression in the endothelial cells. **A**, **B** Representative images illustrate IgG and albumin extravasation in the cerebral cortex of endothelial-specific IRS-1 transgenic rats (eTg/0) and wide type control rats (eWT) at basal state (**B**, i.e. on P7 before HI), and the relative integrated optic density (IOD) of IgG (*N* = 6, *P* = 0.01, *t* = −3.16) and albumin (*N* = 6, *P* = 0.077, *t* = −2.117) signals was quantified. Scale bar: 125um. Independent *t*-test was used for statistical analysis. All data are presented as mean ± SD. * *P* < 0.05. **C** The eTg/0 rats had more perivascular fibrin deposits than that of eWT rats at basal state. *N* = 4, Scale bar: 50um. Inset scale bar: 50 μm (**D**) (Upper panel), The eTg/0 rats had less tight junction protein ZO-1 expression that co-localized with RECA(+) vessels than that of eWT rats at basal state. *N* = 4, Scale bar: 25 μm. (Lower panel) In the eTg/0 rats at basal state (eTg/0-B), in contrast to ZO-1, other tight junction proteins (claudin-5 and occludin) showed more co-localized with RECA (+) vessels. Scale bar: 50 μm. **E** Upregulation of IRS-1 associated with high level of ZO-1 protein expression in the primary endothelial cells (PEC) from eTg/0 pups (PEC-eTg/0) and IRS-1 overexpressed hCMEC clones (hCMEC-IRS-1 C1) compared to PEC from eWT pups (PEC-eWT) and hCMEC control clones (hCMEC-pTag), respectively. *N* = 5. **F** The distribution of ZO-1 became scattered throughout the cytoplasm of hCMEC-IRS-1 cells and PEC-eTg/0 compared to that of hCMEC-pTag control cells and PEC-eWT. *N* = 4, Scale bar: 25 μm.

We next examined whether overexpression of IRS-1 affected the tight junction integrity of the endothelial cells in vivo and in vitro at basal state (i.e. on P7 before HI). The eTg/0 pups had BBB dysfunction as evidenced by significantly increased extravasation of IgG and fibrin (Fig. 4A–C, Supplementary Fig 4) compared to that of the eWT pups. In addition, we found obvious ZO-1 expression in the RECA (+) vessels in the eWT pups, whereas reduced levels of ZO-1 co-localized with the RECA (+) vessels was shown in the eTg/0 pups (Fig. 4D, upper panel). In contrast to ZO-1, other tight junction proteins, claudin-5 and occludin, showed better co-localization with RECA (+) vessels in the eTg/0 (Fig. 4D, lower panel) and eWT pups (Supplementary Fig 3).

Given that overexpression of IRS-1 affected the tight junction integrity of the endothelial cells in vivo at the basal state without HI, we further addressed whether IRS-1 overexpression affected the tight junction integrity of endothelial cells in vitro at the basal level under normoxia. By establishing primary endothelial cell cultures (PEC) from eWT (PEC-eWT) and eTg/0 (PEC-eTg/0) pups, we found higher levels of IRS-1, ZO-1 and occludin protein expressions in PEC-eTg/0 than that in PEC-eWT (Fig. 4E, left panel). The IRS-1 overexpressed hCMEC (human cerebral microvascular endothelial cell) clones (hCMEC-IRS-1 C1, C2) also had higher levels of IRS-1 and ZO-1 proteins than that of the hCMEC control clone (hCMEC-pTag) (Fig. 4E, right panel). The distribution of ZO-1 was located along the cell membrane of cell–cell junctions in the hCMEC-pTag and the PEC-eWT. In contrast, the ZO-1 distribution became dispersed throughout the entire cell in hCMEC-IRS-1 and PEC-eTg/0 (Fig. 4F).

### mTOR activation induced by endothelial IRS-1 overexpression increased vascular permeability and the disassembly of tight junction ZO-1 in vivo and in vitro

**Fig. 5:**
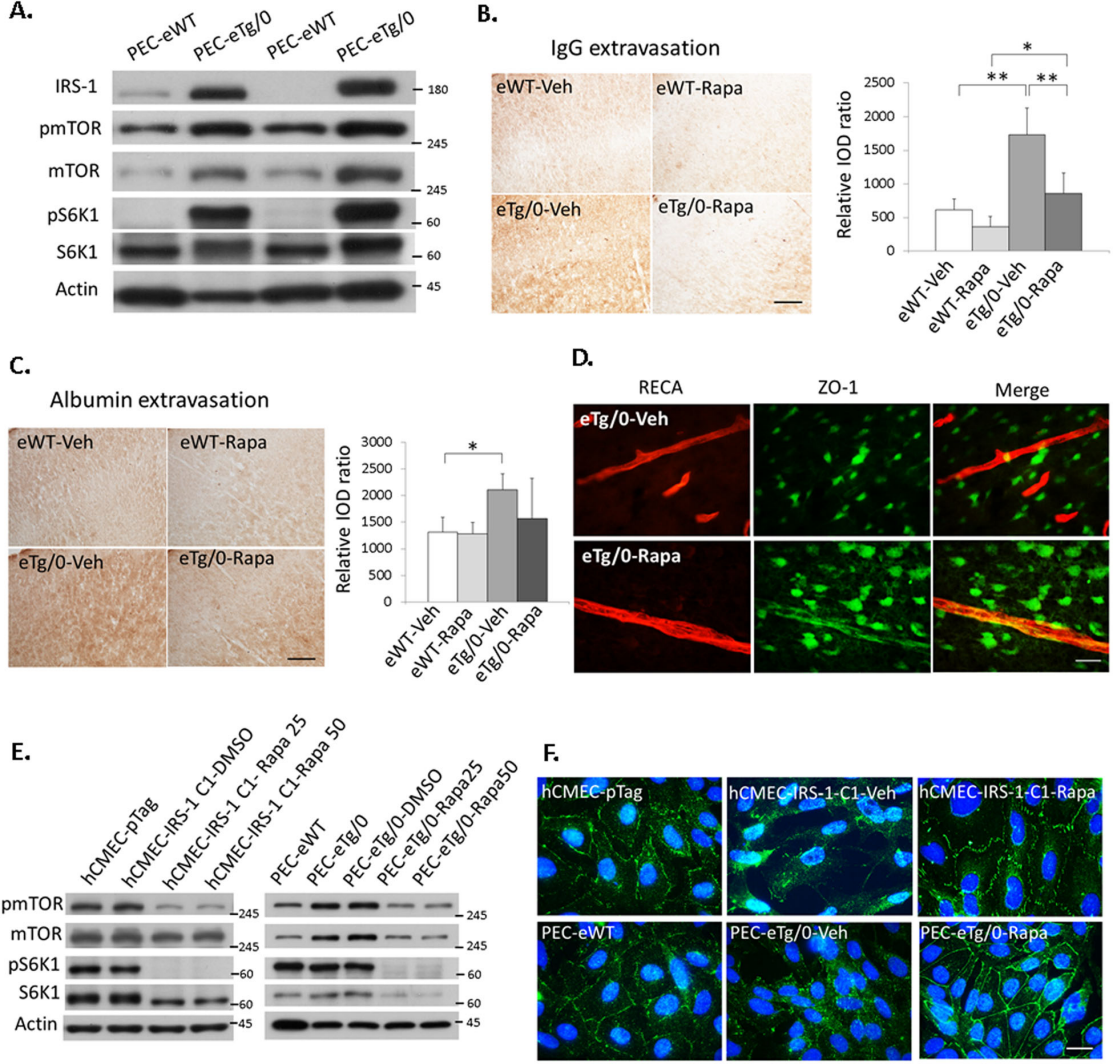
mTOR inhibition reversed the disturbed barrier functions induced by endothelial IRS-1 overexpression. (**A**) The primary endothelial cells from eTg/0 pups (PEC-eTg/0) had higher protein levels of phospho-mTOR (pmTOR), mTOR, phospho-S6K1(pS6K1), and S6K1at basal state (on P7 before HI) than the primary endothelial cells from eWT pups (PEC-eWT) (N = 6). (**B, C**) Representative images illustrate IgG and albumin extravasation at 24 h after HI in the cerebral cortex of eTg/0 and eWT treated with rapamycin (Rapa) or vehicle (Veh), respectively. The relative integrated optic density (IOD) of IgG and albumin signals was quantified. N = 6, Scale bar: 125um. One-Way ANOVA (Scheffe’s post-hoc test) was used for statistical analysis (b: F value = 29.083; c: F value = 4.567). All data are presented as mean ± SD. * *P* < 0.05, ** *P* < 0.001. (**D**) Rapamycin increased ZO-1 expression in the RECA ( + ) vessels in the eTg/0 rats compared to vehicle. Scale bar: 25um. N = 3 (**E**) The expression levels of pmTOR and pS6K1 were suppressed by 25 nM or 50 nM rapamycin in the IRS-1 overexpressed hCMEC clone (hCMEC-IRS-1 C1) (left panel, N = 4) and the PEC-eTg/0 (right panel, N = 4). (**F**) Representative images illustrate the disassembled distribution of ZO-1 in the cytoplasm induced by IRS-1 overexpression in the hCMEC-IRS-1 C1 and the PEC-eTg/0. The distribution of ZO-1 became assembled in the cell membrane by rapamycin (50 nM) treatment. Scale bar: 25 μm. N = 3.

To investigate the possible mechanism involved in endothelial IRS overexpression-mediated increases of the vascular permeability and disassembly of tight junction proteins, we examined the signaling molecules downstream of IRS-1. We found that the PEC-eTg/0 had higher protein levels of phospho-mTOR (pmTOR), mTOR, phospho-S6K1, and S6K1 at basal state (on P7 before HI) than that of the PEC-eWT, indicating that IRS-1 overexpression greatly activated the mTOR pathway (Fig. 5A). In vivo administration of rapamycin, an mTOR inhibitor, significantly decreased IgG (Fig. 5B) but less significantly decreased albumin extravasation in the eTg/0 rats (Fig. 5C). In the eTg/0 pups, rapamycin administration increased ZO-1 expression in the RECA (+) vessels compared to vehicle treatment (Fig. 5D). Rapamycin treatment markedly reduced the expression of mTOR, phospho-mTOR and mTOR-mediated downstream pathways,S6K1 and phospho-S6K1, in the hCMEC-IRS-1 clone and the PEC-eTg/0 compared to vehicle treatment (Fig. 5E). Furthermore, the disorganized distribution pattern of ZO-1 found in the hCMEC-IRS-1 clones and the PEC-eTg/0 became well assembled in the cell membrane at cell-cell junctions after rapamycin treatment (Fig. 5F).

### Rapamycin treatment preserved the loss of tight junction protein, attenuated BBB leakage and neuronal apoptosis, and provided neuroprotection after HI in the endothelial-specific IRS-1 transgenic rats

**Fig. 6:**
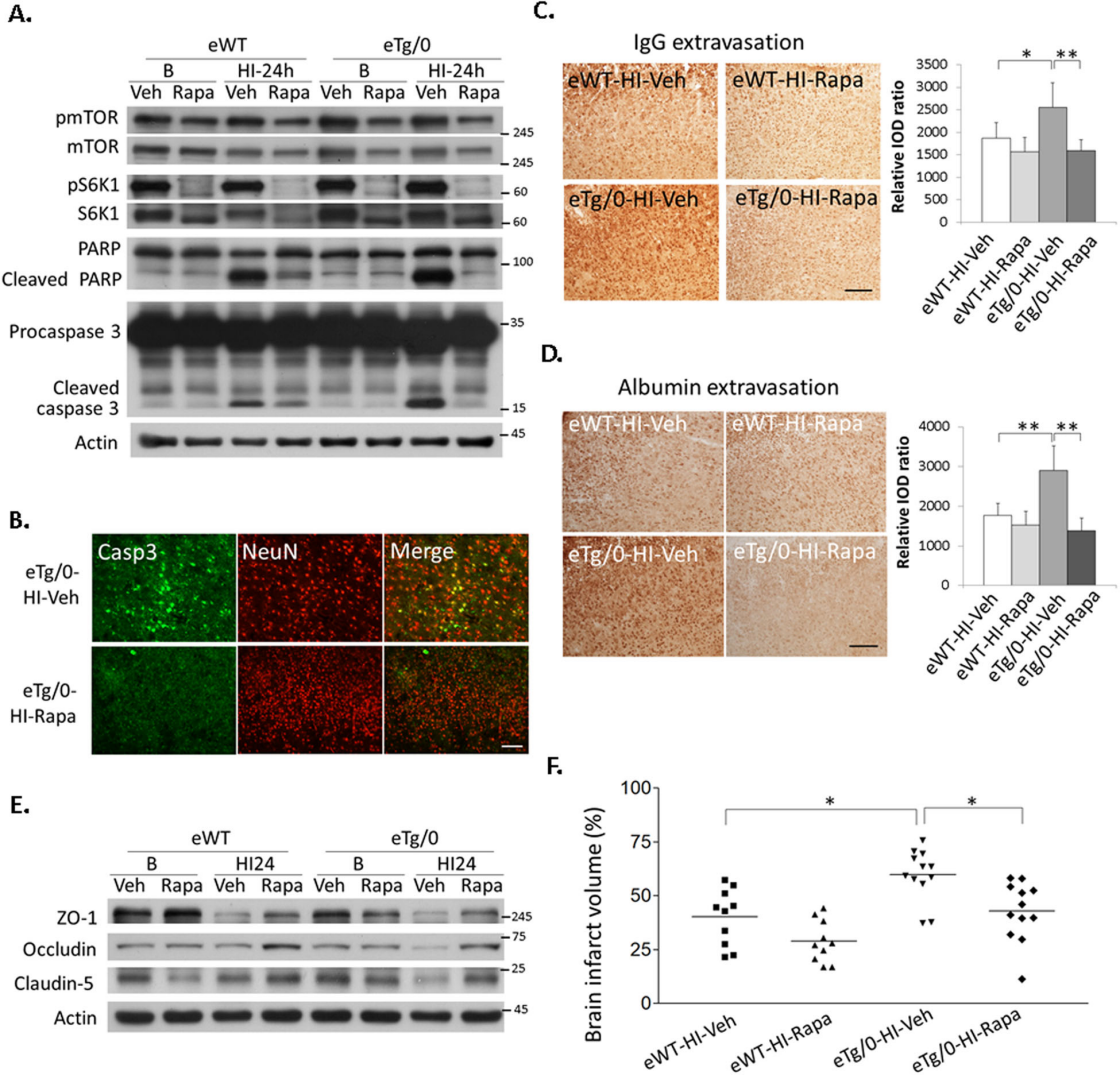
mTOR inhibition reduced HI brain damages in the endothelial-specific IRS-1 transgenic rats. **A** Compared to vehicle, rapamycin treatment greatly decreased the cleaved-PARP and -caspase 3 levels after HI in the eWT and eTg/0 pups. *N* = 4. **B** The cleaved caspase 3 (+) and NeuN (+) apoptotic neurons were reduced in the rapamycin (Rapa)-treated eTg/0 rats than that in the vehicle (Veh)- treated eTg/0 rats at 24 h after HI. Scale bar: 50 μm. *N* = 4. **C**, **D** The extravasation of IgG and albumin in the cerebral cortex at 24 h after HI reduced in the rapamycin-treated eTg/0 pups compared to that of vehicle-treated eTg/0 pups. *N* = 6, Scale bar: 125um. One-Way ANOVA (Scheffe’s post-hoc test) was used for statistical analysis (**C**: *F* value = 8.617; **D**: *F* value = 16.445). All data are presented as mean ± SD. * *P* < 0.05, ** *P* < 0.01. **E** Expression of tight junction proteins, ZO-1, occludin and claudin-5, were relatively preserved by treatment of rapamycin at 24 h after HI compared to vehicle in the eTg/0 and the eWT rats. *N* = 4. **F** The brain infarct volume on P21 (14 days after HI) was quantified. The brain infarct volume was significantly reduced after treatment of rapamycin in the eTg/0 (*N* = 12) or eWT rats (*N* = 10). One-Way ANOVA (Scheffe’s post-hoc test) was used for statistical analysis (*F* value = 11.968). * *P* < 0.05.

Rapamycin treatment reduced levels of pmTOR and its downstream pS6K1 at basal state and also at 24 h after HI in the cortex of the eWT and eTg/0 pups (Fig. 6A). Compared to vehicle, rapamycin treatment greatly decreased levels of the cleaved-PARP and -caspase 3 after HI in the eWT and eTg/0 pups (Fig. 6A). Rapamycin also reduced the number of cleaved-caspase 3 (+) neurons in the cortex in eTg/0 pups (Fig. 6B). Moreover, after HI, the BBB leakage as evidenced by IgG and albumin extravasation was also markedly diminished in the rapamycin-treated eTg/0 pups compared to that of vehicle-treated eTg/0 pups (Fig. 6C, D). The loss of the tight junction proteins, ZO-1, occludin and claudin-5, found in vehicle-treated eWT and eTg/0 pups, was partially restored in ramapycin-treated eWT and eTg/0 pups (Fig. 6E). In addition, rapamycin significantly attenuated the infarct volume in both eWT and eTg/0 pups. Importantly, the brain infarct volume of the rapamycin-treated eTg/0 pups was reduced and similar to that of the vehicle-treated eWT pups (Fig. 6F). Moreover, compared with their own respective vehicle-treated pups, rapamycin-treated eTg/0 pups showed more infarct volume reduction (16.9%) than rapamycin-treated eWT pups (11.3%).

## Discussion

Here, IRS-1 overexpression by intra-cerebroventricular injection of recombinant adenovirus harboring IRS-1 markedly reduced apoptotic neuronal death and attenuated BBB disruption in the neurovascular unit after HI. Moreover, for further translational purposes, transgenic rats that are physiologically and genetically more close to humans than transgenic mice were used. The neuronal IRS-1 transgenic rat pups had attenuated apoptotic neuronal death and decreased BBB disruption in the neurovascular unit, and showed reduced brain damage after HI. In contrast, the endothelial IRS-1 transgenic rat pups showed augmented apoptotic neuronal death, BBB disruption, reduced tight junction proteins in the neurovascular unit, and exaggerated brain damage outcome after HI. Furthermore, our in vivo and in vitro studies found that mTOR overactivation induced by endothelial IRS-1 caused increased vascular permeability and tight junction protein disassembly. Rapamycin treatment preserved the loss of tight junction proteins, attenuated BBB leakage and neuronal apoptosis, and provided significant neuroprotection after HI in the endothelial IRS-1 transgenic pups. Overall, our data demonstrate that neuronal IRS overexpression protects neurovascular integrity and is neuroprotective against HI. In contrast, vascular endothelial IRS-1 overexpression resulted in disrupted tight junction protein assembly, resulted in increased neurovascular damage and worsened brain damage outcome. Thus, IRS-1 overexpression in the neuron and the endothelial cells play opposite roles in the neurovascular integrity after HI.

Insulin signaling in the brain has become an important issue because brain insulin resistance appears to be an early and common feature of many neurodegenerative diseases [19]. The insulin receptor is enriched in the developing brain compared with the adult brain, and also enriched in the neurons and endothelial cells [20, 21]. In addition to regulating glucose metabolism, insulin signaling pathway has pleiotropic effects on the central nervous system [22]. Under stress conditions, insulin signaling restores oxidative stress-induced Akt inactivation, increases gene transcription of Bcl-2, precludes caspase-3 expression, suppresses glutathione peroxidase-1 expression [23–25]. Through activating phosphatidylinositol 3-kinase (PI3K)/Akt, insulin signaling subsequently activates the survival transcription factor CREB, and inhibits GSK-3β and neuronal SAPK [26, 27]. Thus, insulin signaling exerts potent anti-apoptotic effects, suppresses pro-inflammatory cascades and attenuates excitotoxicity under neuronal damage conditions [6, 28]. IRS-1 is expressed in neurons and endothelial cells and is the prominent scaffold protein recruited to the insulin receptor for the downstream signaling. There are more than 20 tyrosine phosphorylation sites of the IRS-1 which recruit proteins equipped with src-homology (SH2) domain, and several enzymes and adapter proteins have been confirmed as partners in IRS-1-mediated signaling cascade [29]. Our previous study and the present study provided evidence that increased IRS-1 levels by intra-cerebroventricular injection AAV harboring IRS-1provided significant neurovascular protection and decreased brain damage after neonatal HI. These findings suggest IRS-1, the key downstream adapter protein of insulin signaling, can be a potential neuroprotective target against brain damage.

The specific features of the BBB, such as tight junctions, play an important role in maintaining brain homeostasis. High-insulin/high-glucose treatment synergistically reduced the tight junction integrity in the hCMEC in an in vitro model of human BBB [30]. We observed increased BBB permeability by selective IRS-1 overexpression in endothelial cells in the eTg/0 rats. In addition to an increase of BBB permeability, the eTg/0 rats showed strikingly disturbed tight junction protein assembly, particularly ZO-1. Observations in the primary endothelial cell cultures of the eTg/0 pups showed that ZO-1 was scattered throughout the cell instead of associated with the membrane as seen in that of the eWT. In support of our finding, in a study of diabetic nephropathy, exposure of rat glomerular epithelial cells to high-glucose levels resulted in a decrease in the intensity of ZO-1 staining and redistribution of ZO-1 from the membrane to the cytoplasm [31]. Here, we showed dysregulated insulin receptor signaling by IRS-1 overexpression in endothelial cells reduced the tight junction integrity through the disturbance of ZO-1 assembly and resulted in increased BBB leakage after HI. Our study indicates that specifically upregulating IRS-1 levels in the endothelial cells worsened the outcome in the integrity of BBB and neurovascular unit after HI.

The mTOR inhibition has been reported to reduce or prevent BBB breakdown in several models of neurological disorders, including cerebral ischemia-reperfusion injury, Alzheimer’s disease, subarachnoid hemorrhage, and status epilepticus [18, 32–37]. The exact mechanisms by which mTOR activation promotes BBB breakdown, however, are still unclear. Studies have suggested mTOR-dependent upregulation of matrix metalloproteinase-9 activity may induce BBB breakdown, and mTOR inhibition maintains the adequate levels of tight junction protein expression [33, 34]. Here, our study showed upregulated mTOR activity by endothelial IRS-1 increased tight junction protein expression, but disturbed tight junction protein ZO-1 distribution. The ZO-1 redistribution and the associated BBB hyperpermeability could be reversed by mTOR inhibition. By reversing BBB hyperpermeability, mTOR inhibition caused more infarct volume reduction in eTg/0 rats than eWT rats. The extra reduction indicated that targeting the endothelium would regulate BBB permeability and reduce ensuing infarct size following HI brain injury.

In addition to the effects on endothelial cells, mTOR has been reported to involve in neuronal apoptosis, autophagy, and neurogenesis [38]. Activating mTOR may limit neuronal death and improve neurological outcomes following ischemic stroke [38]. Paradoxically, mTOR inhibition can either improve or aggravate the brain infarct size following cerebral ischemia in rats [33, 39–41]. Recently, a meta-analysis study concluded that mTOR inhibition by rapamycin reduced brain damage in either neonatal HI or global ischemia, and the efficacy of rapamycin was not affected by the timing of drug administration but depended on the drug dose [42]. Low-dose rapamycin treatment may promote optimal autophagy, and reduce inflammation after cerebral ischemia, and is an effective therapeutic option for ischemic stroke. These neuroprotection effects of low-dose rapamycin were also noted in our rapamycin-treated eWT rats, who reduced the infarct size after HI brain injury compared with vehicle-treated eWT rats.

The findings that IRS-1 upregulation in the endothelial cells exaggerated brain damages after HI may limit its clinical application. It is known that therapeutic agents which are larger than the size limits of BBB (<400 Da), such as chemotherapeutic drugs (MW ~500 Da), neurotrophins (MW ~ 20 kDa), antibodies (MW ~ 150 kDa), and gene vectors (MW ~ 4MDa), cannot pass through the BBB into brain parenchyma because of the tight junction [35, 43–46]. We showed that the endothelial-specific IRS-1 overexpressed transgenic rats had increased BBB permeability of IgG (MW ~ 150 kDa) and fibrin (MW ~ 130 kDa). The enhancement of BBB permeability by endothelial IRS-1 overexpression, however, suggests endothelial IRS-1 as a good candidate for augmenting therapeutic drug delivery across the BBB for the treatment of brain disorders, such as brain tumor or neurodegenerative diseases.

In conclusion, the present study using transgenic rats, which are physiologically and genetically more close to humans than transgenic mice, revealed that differential overexpression of IRS-1 in neurons and in endothelial cells have opposite effects on neurovascular integrity after neonatal HI brain injury. Our findings suggest that developments of neuron-specific therapy augmenting IRS-1 function would be a better choice for rescuing neonatal HIE.

## Methods

### Animal experiments

#### Animals

Sprague-Dawley rats were purchased from BioLASCO Taiwan Co., Ltd. All study rat pups were housed with their dams with a 12/12-h light/dark schedule in a temperature and humidity controlled colony room until weaning on postnatal (P) day 21, and then subsequently housed in groups of 4–5 per cage and fed *ad libitum*. To exclude the effects of gender, only male pups were used in the study. All experimental animals were randomly assigned.

#### Transgene construction and transgenic rat production

A rat IRS-1 cDNA was purchased from Origene and sub-cloned into pCMV-3Tag vector to fuse with 3 × Flag tags at the C-terminus. An IRES-tdTomato fragment excised from vector pLVX-IRES-tdTomato was inserted downstream of IRS-1-3xFlag. A bovine growth hormone polyadenylation (bGH polyA) sequence was then inserted following the IRS-1-3 × Flag-IRES-tdTomato fragment. This expression cassette was then inserted into the start codon of *Tie2* or *Nse* gene separately in individual rat genomic BAC clone (CH230-351N16 for *Tie2* and CH230-409G22 for *Nse*, all from BAC/PAC Resource Center) by a Red/ET DNA Recombineering kit (GeneBridges). The resulting transgene construct based on BAC was linearized, isolated by pulsed-field gel electrophoresis, and purified for microinjected into pronuclei of the Sprague-Dawley rat embryo, which were implanted into the oviducts of pseudopregnant recipients. Pups were screened for the presence of a transgene at 2–3 weeks of age by tail DNA genotyping. Primers used for sequencing IRS-1 and for genotyping were listed in Supplementary Table 1. Transgenic founders are identified by visualizing tdTomato on specific cells (endothelium or neuron) in fixed brain slices and used for breeding to establish transgenic line.

#### Neonatal HI brain injury

On P7, HI brain injury was induced by permanently ligating right common carotid artery followed by hypoxia for 2 h in airtight jars filled with humidified 8% oxygen [17]. During hypoxia, the temperature in the chamber was maintained at 34.5 °C to achieve an average rectal temperature of 36–36.5 °C (nesting temperature) monitored by rectal thermometer (Physitemp Instruments, Inc., Clifton, NJ). After hypoxia, the pups were returned to their dams for recovery and handled every other day.

#### Rat pups transduced with IRS-1 recombinant adenovirus

The pups were intra-cerebroventricularly injected with 3 μL of IRS-1 recombinant or control adenovirus (1.5 × 109 ifu/mL, Applied Biological Materials Inc., Canada) on P5. Intracerebroventricular injection was performed in right cerebral hemisphere (in relation to the bregma: 2.0 mm posterior to, 1.5 mm lateral to, and 2.0 mm beneath the skull surface) using a 30-gauge needle.

#### Rapamycin treatment

In rapamycin-treated group, the pups were intra-peritoneally injected with rapamycin (0.5 mg/kg, #R0395,Sigma-Aldrich) on P4, P5 and P6. The vehicle control group was given with 5%Tween80/5%PEG400/ddH_2_O (5cc/kg) intra-peritoneally at the same time.

#### Outcome measurement

##### Immunofluorescence

Frozen sections were probed with primary antibody in PBS/ 0.01% Triton X-100 at 4 °C overnight. All primary antibodies were listed in Supplementary Table 2. The sections were then incubated with Alexa Fluor 488-conjugated anti-goat IgG or Alexa Fluor 594-conjugated anti-goat IgG (Invitrogen) secondary antibodies for 2 h. Images were acquired on a Nikon E400 fluorescence microscope (Tokyo, Japan). Digitally captured images were analyzed using NIS-Elements imaging software (Nikon, Tokyo, Japan).

##### SDS-PAGE and immunoblotting

Samples were lysed with ice-cold whole cell lysis buffer and homogenized using a sonicator. After the lysates had been centrifuged, the supernatant protein samples (50 μg/lane) were separated by sodium dodecyl sulfate–polyacrylamide gel electrophoresis (SDS-PAGE) and then electrotransferred onto polyvinylidene difuoride (PVDF) membrane. The membrane was subsequently incubated with primary antibody at 4 °C overnight. All primary antibodies were listed in Supplementary Table 3. Thereafter, the membranes were incubated with a HRP-conjugated secondary antibody (1:10000) for 2 h at room temperature. Finally, the membrane was visualized using the Immobilon Western Chemiluminescent HRP Substrate (WBKLS0500, Millipore). Every membrane was stripped and incubated with anti-mouse actin antibody (1:10000) for semi-quantification.

##### BBB damage assessments

*IgG and albumin extravasation*. Brains were cut serially in 20-μm cryosections. Four sections per brain, two at the striatum (0.26 mm and 0.92 mm posterior to the bregma) and another two at the dorsal hippocampus (3.14 mm and 4.16 mm posterior to the bregma) according to a rat brain atlas, were selected for staining. The brain sections were incubated with primary anti-IgG antibody (HRP-conjugated 1:200; AP136P, Chemicon) or anti-albumin antibody (1:200, Genetex) for 2 h at room temperature. Biotin-peroxidase signals were detected using 0.5 mg/mL 3′3′-diaminobenzidine (DAB)/0.003% H_2_O_2_ as a substrate and were microscopically recorded (Eclipse E400, Nikon). The integrated optic density of IgG and albumin signals were further analyzed and the averaged value at three visual fields in the cortex per sections at ×200 magnification (0.145 mm^2^/per visual field).

*Fibrin leakage*. Brain cryosections were probed with primary antibodies, anti-fibrinogen (1:200 Dako) and anti-RECA antiboties (1:100 Abcam) as described in the previous immunefluorescence method.

##### Infarct volume measurement

Fourteen days after HI, brains were obtained, embedded in paraffin blocks, and sectioned coronally (10 μm thick). One in every 20 sections was stained with cresyl violet (Nissl stain), and at least 13 sections per rat were used to measure the brain volume loss. Brain-volume loss in the lesioned versus the non-lesioned cerebral hemisphere was calculated: (non-lesioned hemisphere volume−lesioned hemisphere volume)/(non-lesioned hemisphere volume).

### Cellular experiments

#### Human cerebral microvascular endothelial cell line (hCMEC) and IRS-1 overexpressing clone

Human Cerebral Microvascular Endothelial Cell (hCMEC) were purchased from Cellutions Biosystems Inc.(Ontario, Canada) and were passed in endothelial cell growth medium EndoGRO-MV Complete Culture Media Kit (Millipore) supplemented with human basic fibroblast growth factor (Sigma) and penicillin-streptomycin (Life technologies) [47]. To generate stable IRS-1-overexpressing hCMEC, a vector pTagRFE-N harboring IRS-1cDNA was transfected into 5 × 10^5^ hCMEC cells using lipofectamine 2000 reagent (Invitrogen) at 80% confluence for 48 h. Different clones with IRS-1 overexpression were isolated after G418 selection (500ug/mL, Gibco) of the transfected cells over two weeks, and validated by immunoblotting.

#### Primary endothelial cells from transgenic rat

Four 2-week-old IRS-1 transgenic SD rats and control rats were anesthetized with ether and were sacrificed by decapitation. Brain tissues were minced and gently dissociated in buffer containing 15 mM Hepes (pH 7.4), 153 mM NaCl, 5.6 mM KCl, 2.3 mM CaCl2x 2H_2_O, 2.6 mM MgCl_2_*x*6H2O, and 1% Bovine Serum Albumin (BSA). The resulting microvessel fraction was then sequentially digested with collagenase/dispase at a concentration of 1 mg/mL (Sigma-Aldrich) for 45 min at room temperature. After centrifugation, the pellet containing primary endothelial cells (PEC) was resuspended in Dulbecco’s modification of Eagle’s medium (DMEM, GIBCO) supplemented with 10% fetal bovine serum (FBS), 1% L-glutamine and 4 μg/ml puromycin in a humidified incubator (37 °C, 5% CO2). Cells from passage 2 to 5 were used.

#### Rapamycin treatment

In rapamycin-treated group, hCMEC IRS-1 clones and PEC were treated with 25 nM or 50 nM rapamycin (#1292, Tocris) for 72 h in their culture medium. DMSO-treated group (#D2650, Sigma) was used as a vehicle control.

### Statistics

A commercial program (SPSS version 20.0; SPSS Institute, Chicago, IL) was used for the statistical analysis. Data were presented as means ± standard deviation (SD). Each dataset was tested for normal distribution first (Shapiro–Wilk test). Independent *t*-test was applied if the data were normally distributed. In the case of multiple comparisons, One-Way ANOVA plus Scheffe’s post-hoc test was applied. Statistical significance was set at a two-tailed p < 0.05 and all probabilities were 2-tailed. The statistician was blinded to the group allocation.

## Supplementary information

Supplementary Figure 4

Supplementary figure legends

supplementary Figure 4

Supplementary Figure 1

Supplementary Figure 2

Supplementary Figure 3

data set

## Data Availability

The datasets generated and analyzed during the current study are available from the corresponding author upon reasonable request.
