# Supplementary figures and images for: Endothelial-specific insulin receptor substrate-1 overexpression worsens neonatal hypoxic-ischemic brain injury via mTOR-mediated tight junction disassembly
Source: Cell Death Discov. 2021 Jun 29;7:150. doi: 10.1038/s41420-021-00548-3 (PMC8257791; doi:10.1038/s41420-021-00548-3)

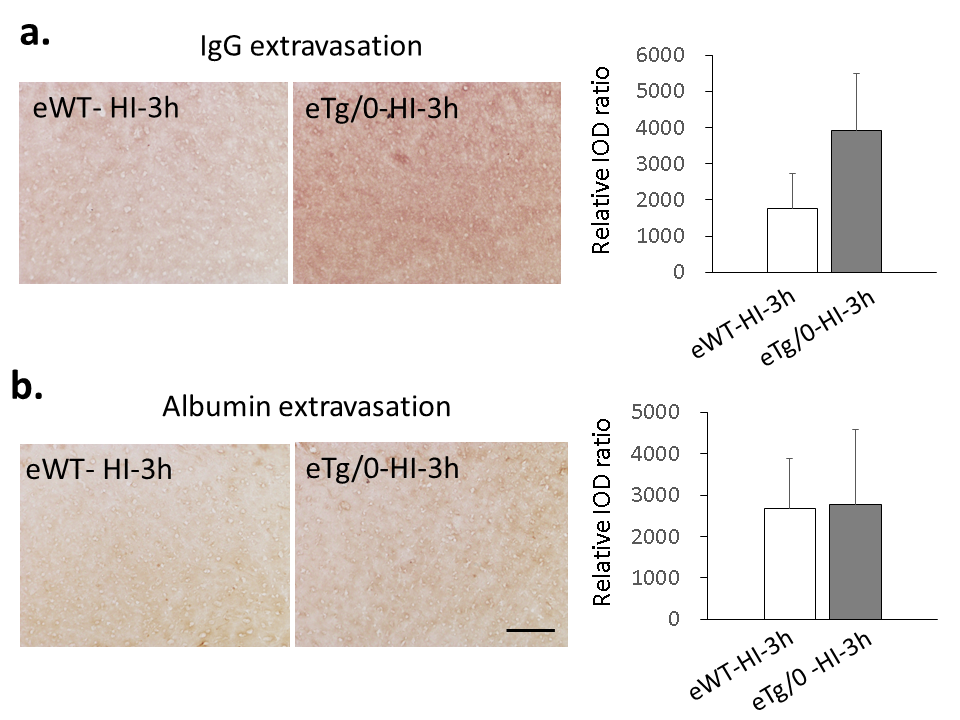

Supplement: Supplementary file 1 — Supplementary Figure 4 [file 41420_2021_548_MOESM1_ESM.tif]

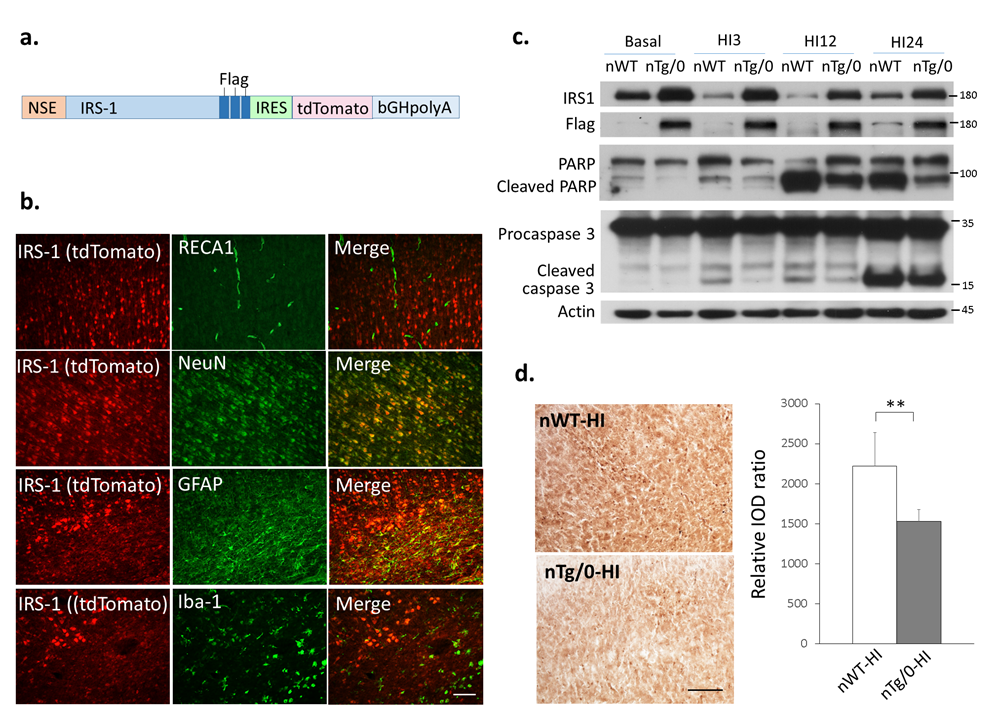

Supplement: Supplementary file 4 — Supplementary Figure 1 [file 41420_2021_548_MOESM4_ESM.tif]

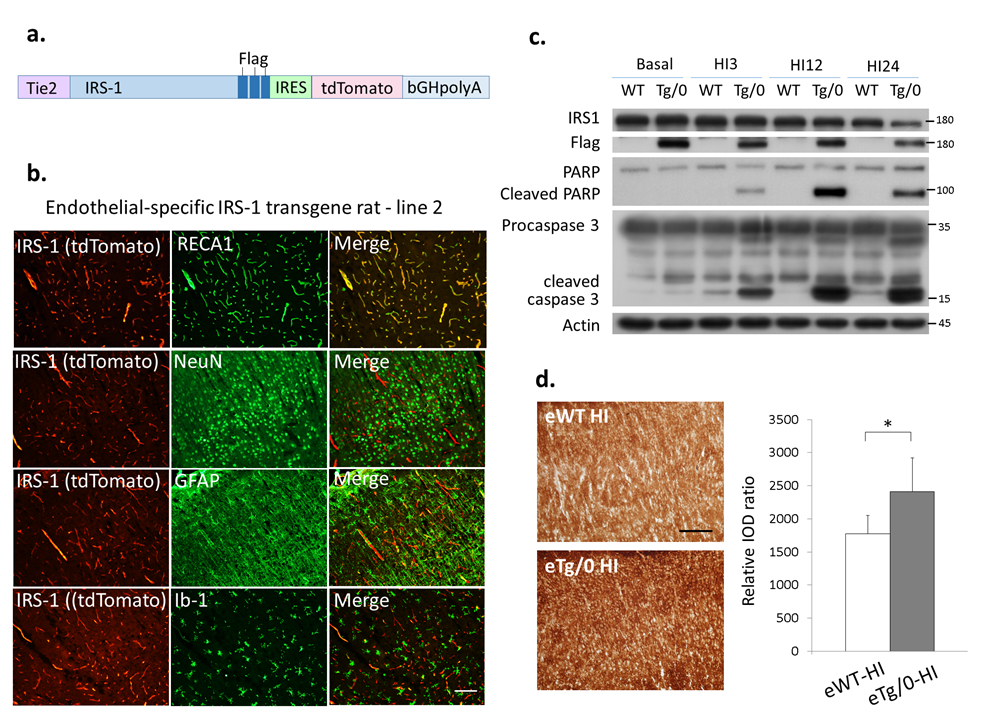

Supplement: Supplementary file 5 — Supplementary Figure 2 [file 41420_2021_548_MOESM5_ESM.tif]

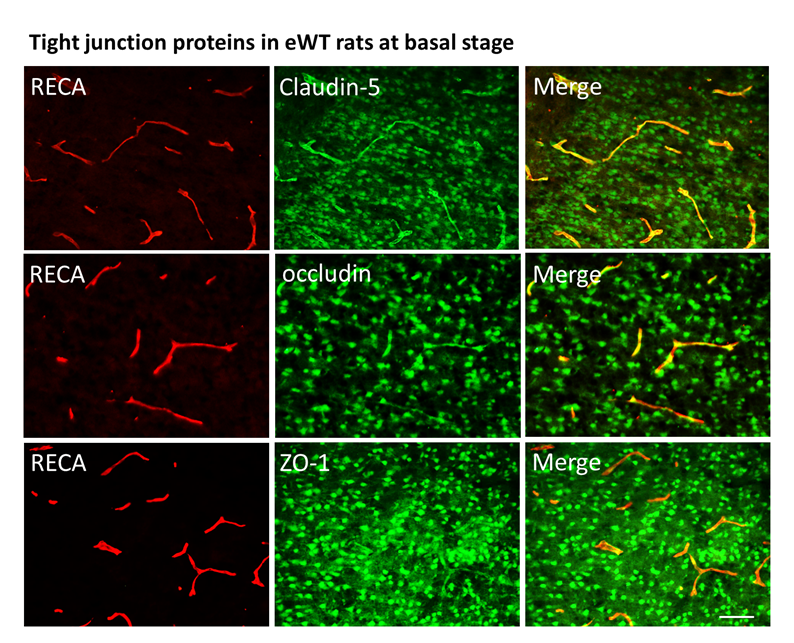

Supplement: Supplementary file 6 — Supplementary Figure 3 [file 41420_2021_548_MOESM6_ESM.tif]
