# Supplementary material for: Endothelial-specific insulin receptor substrate-1 overexpression worsens neonatal hypoxic-ischemic brain injury via mTOR-mediated tight junction disassembly
Source: Cell Death Discov. 2021 Jun 29;7:150. doi: 10.1038/s41420-021-00548-3 (PMC8257791; doi:10.1038/s41420-021-00548-3)
Supplement: Supplementary file 2 — Supplementary figure legends [file 41420_2021_548_MOESM2_ESM.docx]

**Supplementary Figure legends**

**Supplementary Figure 1.**

Another line of Neuron-specific IRS-1 transgenic rats had also reduced neurovascular injury and brain damage after HI

(**a**) Diagram of the transgene which contains neuron-specific promoter (NSE), full-length IRS-1 tagged with 3x Flag and IRES-directed tdTomato. (**b**) The IRS-1/tdTomato were expressed specifically on NeuN (+) neurons, and not on RECA(+) endothelium cells, GFAP(+) astrocytes or Iba-1(+) microglia in the cerebral cortex of P7 rat pups. Scale bar: 50 μm. (**c**) The neuron-specific IRS-1 transgenic (nTg/0) rats showed upregulated IRS-1 in the brain, and reduced cleaved-PARP, and –capsease 3 after HI compared to the wild-type littermates (nWT) rats. N=3 (**d**) Representative images illustrate immunoglobulin G (IgG) extravasation in the cerebral cortex at 24 hours after HI between the nTg/0 than nWT rats, and the relative integrated optic density (IOD) of IgG signal was quantified and compared between groups. Scale bar: 125um. The data are presented as mean ± SD. ** P < 0.01

**Supplementary Figure 2.**

Another line of Endothelial-specific IRS-1 transgenic rats also showed augmented neurovascular injury and brain damage after HI

(**a**) Diagram of the transgene containing endothelial-specific promoter (Tie2), full-length IRS-1 tagged with 3x Flag and IRES-directed tdTomato. (**b**) IRS-1/tdTomato was expressed specifically on RECA (+) endothelium cells, and not on NeuN(+) neurons, GFAP(+) astrocytes or Iba-1(+) microglia in the cerebral cortex of P7 rat pups. Scale bar: 50 μm. (**c**) The endothelial-specific IRS-1 transgenic (eTg/0) rats had increased levels of cleaved-PARP, and –capsease 3 after HI compared to the wild type littermates (eWT) rats. N=3 (**d**) Representative images illustrate immunoglobulin (IgG) extravasation in the cerebral cortex at 24 hours after HI between eTg/0 and eWT rats, and the relative integrated optic density (IOD) of IgG signal was quantified. Scale bar: 125um. The data are presented as mean ± SD. * P < 0.05.

**Supplementary Figure 3.**

In the wild type littermates (eWT) of endothelial-specific IRS-1 transgenic rats, the tight junction associate proteins including claudin-5, occluding and ZO-1 mainly co-localized with RECA(+) vessels. Scale bar: 50 μm

**Supplementary Figure 4.**

Representative images illustrate IgG and albumin extravasation in the cerebral cortex at 3 hours after HI between eTg/0 and eWT rats, and the relative integrated optic density (IOD) of IgG and albumin signal were quantified. Scale bar: 125um. N = 4. The data are presented as mean ± SD.
